# Natural Killer Cell Memory: Progress and Implications

**DOI:** 10.3389/fimmu.2017.01143

**Published:** 2017-09-13

**Authors:** Hui Peng, Zhigang Tian

**Affiliations:** ^1^Institute of Immunology, The CAS Key Laboratory of Innate Immunity and Chronic Disease, School of Life Sciences and Medical Center, University of Science and Technology of China, Hefei, China; ^2^Collaborative Innovation Center for Diagnosis and Treatment of Infectious Diseases, State Key Laboratory for Diagnosis and Treatment of Infectious Diseases, First Affiliated Hospital, College of Medicine, Zhejiang University, Hangzhou, China

**Keywords:** natural killer cell, immunological memory, contact hypersensitivity, cytomegalovirus infection, therapeutic potential

## Abstract

Immunological memory is a cardinal feature of adaptive immunity. Although natural killer (NK) cells have long been considered short-lived innate lymphocytes that respond rapidly to transformed and virus-infected cells without prior sensitization, accumulating evidence has recently shown that NK cells develop long-lasting and antigen-specific memory to haptens and viruses. Additionally, cytokine stimulation alone can induce memory-like NK cells with longevity and functional competence, leading to emerging interest in harnessing NK cell memory for cancer immunotherapy. Here, we review the evidence of NK cell memory in different settings, summarize recent advances in mechanisms driving the formation of NK cell memory, and discuss the therapeutic potential of NK cells with memory-like properties in the clinical setting.

## Introduction

The immune system has the ability to remember a previous encounter with an antigen and mount a stronger response with faster kinetics upon reencountering the same antigen. This phenomenon is known as immunological memory, which is the theoretical basis for vaccination (1, 2). Immunological memory is considered a cardinal feature of adaptive immunity, as adaptive lymphocytes are highly specific to a particular antigen due to somatic diversification mechanisms. Both T and B cells express recombination-activating genes (RAGs) that mediate the rearrangement of genes encoding antigen recognition receptors, thereby enabling the generation of antigen receptor diversity. Following initial exposure to an antigen, antigen-specific T and B cells undergo clonal expansion. Upon antigen clearance, most of these effector cells die *via* apoptosis during the contraction phase, but some survive and progressively differentiate into long-lived memory cells that mediate a faster and more robust antigen-specific response than naïve cells (3, 4).

Unlike adaptive immunity, the innate immune system mounts a rapid response against pathogens and transformed cells in the absence of prior sensitization (5). Innate immune cells do not express rearranged antigen receptors but rely on a set of germ line-encoded receptors to recognize targets. The innate immune system contains numerous distinct cell types, among which natural killer (NK) cells have long been considered short-lived and aspecific effector cells (6). NK cells were originally identified in 1975 based on their spontaneous ability to lyse tumor cells without prior sensitization (7). It is now clear that another important function of NK cells is the production of multiple cytokines, such as interferon-γ (IFN-γ), early in an immune response (8, 9). NK cell effector functions are under the control of a complex array of surface receptors, delivering either inhibitory or activating signals (10). Since their discovery, abundant evidence has highlighted the importance of NK cells in host defense against infections and tumors (11–14) and in modulating adaptive immune responses through both direct interactions with T cells and indirect mechanisms, such as the induction of dendritic cell (DC) maturation (15–18).

During the past decade, however, increasing evidence has shown that NK cell-mediated immune responses share common features with adaptive immunity, and NK cells acquire immunological memory in a manner similar to T and B cells (19). Here, we summarize recent findings concerning the roles of antigen-specific memory NK cells in contact hypersensitivity (CHS) responses and viral infections and discuss the recent progress in cytokine-induced memory-like NK cell responses in mice and humans, with an emphasis on their potential implications for clinical therapies.

## NK Cell Memory in CHS

**Figure 1:**
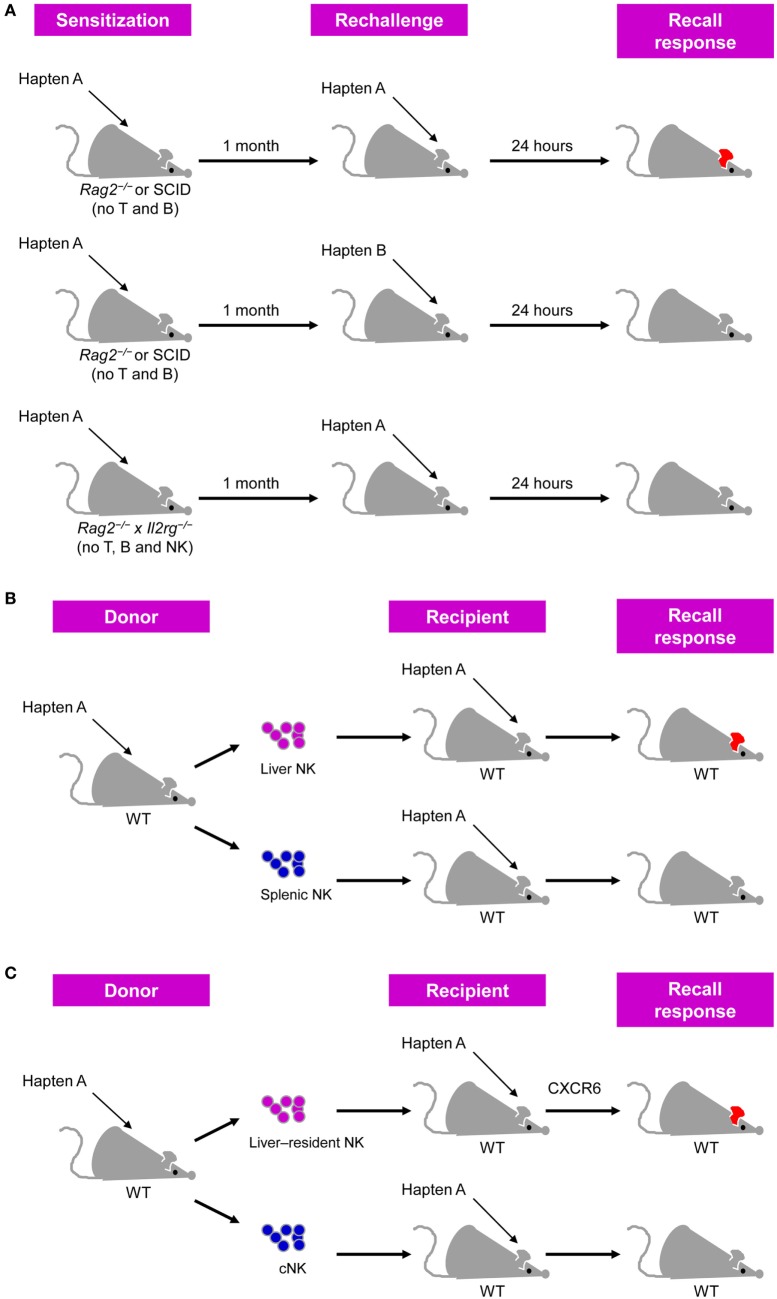
Natural killer (NK) cells confer antigen-specific contact hypersensitivity (CHS) memory responses. **(A)** T cell- and B cell-deficient *Rag2^−/−^* or severe combined immunodeficiency (SCID) mice sensitized by the painting of their skin with a specific hapten developed vigorous CHS upon challenge with the same hapten, but not an unrelated hapten, on their ears. This antigen-specific CHS response did not occur in *Rag2^−/−^* × *Il2rg^−/−^* mice lacking T, B, and NK cells. CHS response was determined by measuring ear swelling [adapted from Ref. (27) with permission from Nature Publishing Group]. **(B)** Liver NK cells, but not splenic NK cells, from hapten-sensitized mice transfer hapten-specific memory into naïve recipients. **(C)** Liver-resident NK cells, but not conventional NK (cNK) cells, from hapten-sensitized mice transfer hapten-specific memory into naïve recipients, and this process is dependent on CXCR6.

Antigen-specific memory NK cell responses were first observed in a murine model of hapten-induced CHS (20). This model was established through sensitization *via* painting a specific hapten, such as 2,4-dinitrofluorobenzene (DNFB) or oxazolone (OXA), on mouse skin and subsequent challenge with the same hapten on the ears of the mice, after which the recall responses to the haptens were measured based on ear swelling. CHS responses were previously considered to be primarily mediated by T cells (21, 22), among which αβ T cells are the critical effectors (23), although γδ T cells, NKT cells, and B-1 cells are also involved in this process (24–26). However, von Andrian et al. recently observed hapten-induced CHS in immunodeficient mice lacking T and B cells, such as RAG2-deficient mice and severe combined immunodeficiency (SCID) mice (20). Moreover, NK cell accumulation was observed in the inflamed ears in this model, and depleting NK cells from these immunodeficient mice or using *Rag2^−/−^* × *Il2rg^−/−^* mice lacking NK cells and adaptive lymphocytes resulted in a failure to mount CHS responses (Figure 1A), providing evidence that NK cells can confer antigen-specific memory responses (20).

Interestingly, further analysis showed that the adoptive transfer of hepatic NK cells, but not splenic NK cells, from DNFB-sensitized mice led the recipient mice to develop a CHS response following challenge with DNFB (20) (Figure 1B). A subsequent study further validated the ability of hepatic NK cells transferring DNFB-specific memory (28). In these studies, NK cells were defined as DX5^+^CD3^−^ cells and whether DX5^−^ NK cells possess memory potential in CHS models was not investigated (20, 28). Furthermore, hepatic NK cells expressing the lectin-type receptor Ly49C/I were more potent in transferring CHS responsiveness than hepatic Ly49C/I^−^ NK cells (20). Moreover, the activating receptor NKG2D and adhesion molecules such as CD18, L-selectin, P-selectin, and E-selectin may be involved in the different phases of NK cell-mediated CHS, as antibody-mediated blocking of such molecules in RAG2-deficient mice was shown to suppress hapten-induced CHS responses (20). In addition, liver Thy1^+^Mac-1^+^CD27^−^ NK cells transferred DNFB-specific memory more efficiently than Thy1^+^CD27^−^ NK cells or Thy1*^+^Itgam* (Mac*-*1)^−^*^/^*^−^ NK cells (28). However, as splenic NK cells also express these surface markers mentioned above, these proteins are unlikely to be responsible for the preferential localization of hapten-specific memory NK cells in the liver.

Notably, subsequent studies have revealed that NK cell-mediated hapten-specific CHS responses require CXCR6 (29), a CXCL16 chemokine receptor constitutively expressed on the hepatic sinusoidal endothelium (30). The increased expression of CXCR6 from hepatic NK cells compared with that of splenic NK cells and the tissue-restricted expression of CXCL16 may explain why memory NK cells are concentrated in the liver, rather than in other organs (29, 31). Furthermore, hepatic CXCR6^+^ NK cells can also mount memory responses to viral antigens, including inactivated vesicular stomatitis virus (VSV) and virus-like particles that contain viral proteins from human immunodeficiency virus (HIV) as well as influenza, in a manner similar to that of chemical haptens (29).

Cytokine signaling is also involved in hepatic NK cell-mediated CHS. IL-12, IFN-α, and IFN-γ have been implicated in NK cell-mediated CHS responses to DNFB, whereas the type 2 cytokines IL-4 and IL-13 are dispensable for this process (28). A recent study showed that in a CHS model induced using the pro-hapten monobenzone, which forms a quinone-hapten *via* interactions with the melanosomal enzyme tyrosinase in pigmented cells (32), memory NK cell formation required NLRP3 inflammasome activation in tissue-resident macrophages and its target cytokine, IL-18 (33). Upon skin exposure to monobenzone, tissue-resident macrophages migrated into the draining lymph nodes in an inflammasome-dependent manner, and this effect coincided with local NK cell activation (33), highlighting the importance of an inflammatory milieu for the induction of memory NK cells. Moreover, previous sensitization with monobenzone enhanced NK cell cytotoxicity against B16 melanoma cells or the pigmented melanoma cell line HCmel3 (33), providing evidence of the potential roles of memory NK cells in cancer immunotherapy.

Recent studies in subsets of liver NK cells have revealed that hapten-induced NK cell memory responses are concentrated in a liver-resident NK cell subset phenotypically characterized as CD49a DX5^−^NK1.1^+^Lin^−^. CD49a and DX5 are mutually exclusively expressed on murine liver NK cells and can be used to distinguish two subsets of these cells with distinct phenotypes and functional and transcriptional properties (31, 34). CD49a^−^DX5^+^ NK cells, also known as conventional NK (cNK) cells, are widely distributed and circulate freely throughout the body (31, 35), whereas liver CD49a^+^DX5^−^ NK cells are tissue-resident cells and are dependent on the transcription factors T-bet and Hobbit, but not Eomes, which is not important for cNK cell development (35–37). Consistent with previous findings of CXCR6-dependent NK cell memory responses to haptens (29), CXCR6 was found to be highly expressed on liver-resident NK cells, but nearly absent on cNK cells, which might explain why cNK cells are less efficient in transferring CHS responsiveness than liver-resident NK cells in OXA- or FITC-induced CHS models (Figure 1C) (31). Accordingly, the Kaplan group showed that liver-resident NK cells, rather than cNK cells, could effectively mediate inflammatory skin responses, and this process was inhibited by Langerhans cells (38). Another follow-up study from the Sunwoo group reported that aryl hydrocarbon receptor (AhR)-deficient mice, which show a significant reduction of liver-resident NK cells, exhibit impaired NK cell-mediated CHS (39), emphasizing the importance of liver-resident NK cells in NK cell memory responses to haptens.

Although progress has been made in the field of NK cell memory in CHS, some important issues remain unresolved. As NK cells lack the ability to somatically rearrange their receptors, how these cells distinguish different haptens remains an open question. Furthermore, as CD49a^+^DX5^−^ NK cells rarely circulate or emigrate from the liver at steady state, the mechanism by which memory NK cells migrate from their initial priming site to the liver and eventually to the challenge site is also worthy of in-depth study. In addition, recent studies have shown that, similar to mice, the livers of humans contain a relatively high proportion of CXCR6^+^ NK cells compared to the peripheral blood (40–42); however, these cells express Eomes rather than T-bet, which is in contrast to murine liver-resident NK cells. Nevertheless, the phenotypic similarities between murine and human NK cells raise an interesting question of whether the findings regarding hapten-specific memory mediated by liver NK cells can be translated from mice to humans.

## NK Cell Memory in Viral Immunity

In addition to their memory responses to haptens, NK cells have also been found to confer long-lived, antigen-specific memory responses to viruses (19). NK cell memory in response to viral infections has been described in several species, including mice (29, 43, 44), rhesus macaques (45), and humans (46, 47). Among the various viral infection models, much of the knowledge of NK cell memory has been obtained through murine cytomegalovirus (MCMV) infection, a generally accepted model for the study of human cytomegalovirus (HCMV) (48).

### NK Cell Memory in Response to MCMV

Early studies have indicated a critical role for the activating receptor Ly49H in NK cell-mediated resistance to MCMV infection (49–51). Approximately 50% of splenic NK cells in naïve C57BL/6 mice express Ly49H, which specifically recognizes the MCMV-encoded protein m157. During the first week of MCMV infection, Ly49H^+^ NK cells expand to account for >80% of the total NK cell population, and their expansion is antigen specific, as infection with mutant MCMV lacking m157 or vaccinia virus does not induce preferential proliferation of the Ly49H^+^ subset (43, 51, 52). The Lanier group performed adoptive transfer of Ly49H^+^ NK cells into recipients lacking Ly49H^+^ NK cells (such as mice deficient in DAP12, which transduces signals from Ly49H) or Ly49H-deficient mice to trace the kinetics of these cells in response to MCMV infection, and the results showed that the donor Ly49H^+^ NK cells underwent profound expansion (100- to 1,000-fold), specifically driven by Ly49H-m157 engagement (43). The expansion phase was followed by the contraction of effectors, and the remaining Ly49H^+^ cells could persist in tissues for several months, forming a long-lived memory NK cell pool (43). These self-renewing NK cells exhibited a more mature phenotype and more robust effector functions *ex vivo* than their naïve counterparts. Importantly, these NK cells could undergo secondary or even tertiary expansion after several rounds of adoptive transfer and MCMV infection and mediated more efficient protective immunity against MCMV than naïve Ly49H^+^ NK cells (43, 53). Thus, similar to the adaptive responses of T and B cells, Ly49H^+^ NK cells go through four phases during MCMV infection: expansion, contraction, memory maintenance, and recall responses.

**Figure 2:**
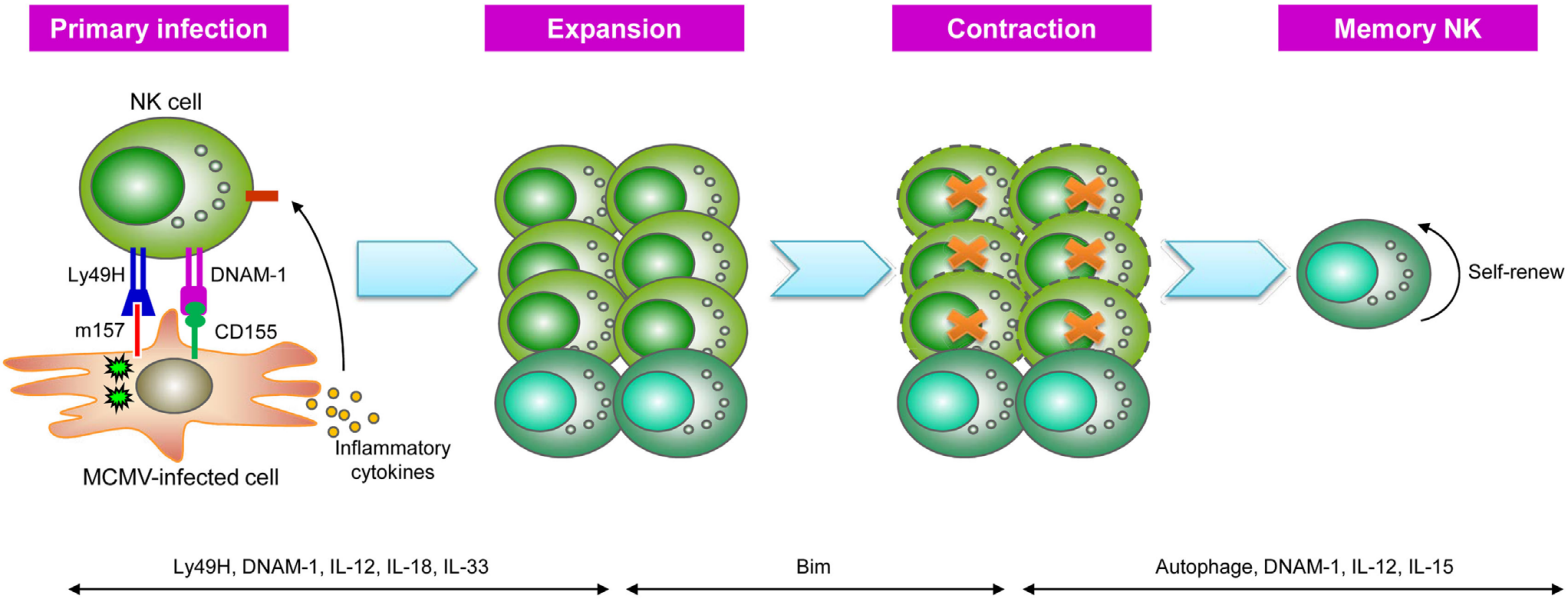
Generation of natural killer (NK) cell memory during murine cytomegalovirus (MCMV) infection. During MCMV infection, NK cells recognize MCMV-infected cells *via* Ly49H-m157 interactions, and Ly49H^+^ NK cells undergo clonal-like expansion, partially driven by the signal mediated by the costimulatory molecule DNAM-1 and pro-inflammatory cytokines, such as IL-12, IL-18, and IL-33. Following NK cell expansion, Ly49H^+^ NK cells enter a contraction phase that is dependent on the proapoptotic factor Bim and subsequently become long-lived memory NK cells. Autophagy, DNAM-1, and IL-12 are important for the generation of memory NK cells, whereas their survival is dependent on IL-15 [adapted from Ref. (19) with permission from Nature Publishing Group].

Subsequent studies have provided further insight into the precise mechanisms underlying MCMV-induced memory NK cell generation (Figure 2). The costimulatory molecule DNAM-1 is required for the expansion of effector Ly49H^+^ NK cells and the differentiation of memory Ly49H^+^ NK cells through the downstream signaling components Fyn and PKCη (54). Moreover, a recent study showed that some proportion of NK cells transiently expressed *Rag1* during development, and RAG deficiency resulted in impaired expansion and persistence of NK cells following MCMV infection (55). Furthermore, cytokine signaling plays important roles in virus-specific NK cell responses at different stages following MCMV infection (56). The IL-12-STAT4 signaling axis is indispensable not only for the proliferation of MCMV-specific NK cells but also for the generation of long-lived memory NK cells (57). IL-18 and IL-33 are specifically required for the optimal expansion of Ly49H^+^ NK cells (58, 59), whereas IL-15 is a key survival factor in NK cells and has been found to be more critical during the maintenance of NK cell memory (60). Further studies revealed that inflammatory cytokines could drive NK cells to express microRNA-155 (miR-155) and Zbtb32 (61, 62), which in turn induce NK cell proliferation *via* antagonizing the antiproliferative factor Blimp-1 (62). Effector cell contraction is also a critical event during the development of long-lived memory cells, and NK cell contraction is dependent on the proapoptotic factor Bim (63). However, the mitochondrial-associated proteins BNIP3 and BNIP3L promote the removal of mitochondria-associated reactive oxygen species and dysfunctional mitochondria *via* autophagy, which serves as a pro-survival mechanism for MCMV-specific NK cells during the effector-to-memory phase transition (64). These findings are consistent with the results of studies revealing the importance of autophagy in memory CD8^+^ T cell formation (65), although the underlying mechanisms of these adaptive lymphocytes remain unknown.

### NK Cell Memory in Response to HCMV

Human cytomegalovirus is a ubiquitous virus associated with high-risk morbidity in immunologically suppressed and immunodeficient individuals (66, 67). In humans, an increased frequency of NK cells expressing the activating heterodimeric receptor CD94-NKG2C has been observed in healthy HCMV-seropositive individuals compared with that in HCMV-seronegative individuals (68–70). Although expansion of the NKG2C^+^ NK cell population has also been observed in patients infected with other viruses, such as HIV (71), hantavirus (72), hepatitis B virus, or hepatitis C virus (47), an increased frequency of NK cells expressing CD94-NKG2C has been observed only in individuals with HCMV coinfection. Moreover, HCMV-infected cells can promote the expansion of NKG2C^+^ NK cells *in vitro*, and the observed cell expansion can be abrogated using a CD94-specific blocking antibody (73). Strikingly, in a case study of a T cell-deficient infant with acute HCMV infection, more than 80% of NK cells were found to express NKG2C with increasing viral loads (74). Analogous to the kinetics of murine Ly49H^+^ NK cells during MCMV infection, NKG2C^+^ NK cells undergo contraction after control of HCMV infection but persist at a relatively high frequency in solid-organ transplant recipients with HCMV viremia (46). Moreover, HCMV reactivation in hematopoietic cell transplantation patients is correlated with the preferential expansion of NKG2C^+^ NK cells exhibiting a mature NK cell phenotype, showing progressive acquisition of CD57 and more potent IFN-γ production than NKG2C^+^ NK cells obtained from seronegative donors (75–77). Thus, the expanding NKG2C^+^ NK cells in HCMV-infected individuals potentially represent a human NK cell population with memory-like properties.

Several recent studies have provided further insight into the mechanisms involved in NK cell memory-like properties driven by HCMV infection. Similar to the requirement of inflammatory cytokines for Ly49H^+^ NK cell responses against MCMV infection, IL-12 produced by CD14^+^ monocytes is also required for the expansion of NKG2C^+^ NK cells in response to HCMV infection (78). Studies have demonstrated the demethylation of conserved non-coding sequence (CNS) 1 of the *IFNG* locus in expanding NKG2C^+^ NK cells, which remains stably imprinted in progeny, similar to memory Th1 cells (79). Furthermore, FcεRIγ-negative NK cells have also been associated with a history of HMCV infection and high expression of NKG2C and CD57 (80–83). These NK cells exhibit more robust antibody-dependent responsiveness through cross-linking of CD16 (FcγRIIIa) than FcεRIγ-expressing NK cells (80, 81). Deficiency of multiple proteins, such as the transcription factor PLZF and the signaling molecules DAB2 and EAT-2, has been observed in these FcεRIγ-negative NK cells, and DNA hypermethylation occurs at the promoter regions of several of these genes (82, 83), further suggesting epigenetic modifications of HCMV-driven memory-like NK cells.

Nevertheless, although numerous studies have provided evidence that the expansion of NKG2C^+^ NK cells may be specific to HCMV infection, there is no direct evidence suggesting that NKG2C^+^ NK cells specifically exert their functions against HCMV infection and mount a recall response to HCMV reactivation. Moreover, it remains unknown whether NKG2C directly recognizes the HCMV protein, similar to the Ly49H-MCMV m157 interaction. In fact, emerging evidence has revealed that HCMV-induced NK cells with adaptive features can occur in the absence of NKG2C (84–86). Thus, the nature of the memory-like responses of human NK cells induced after HCMV infection requires further investigation.

### NK Cell Memory during Other Viral Infections

In addition to CMV, NK cell memory has also been observed in response to other viral infections. Paust et al. showed that murine hepatic NK cells could remember prior encounters with influenza virus, VSV, and HIV and confer virus-specific delayed-type hypersensitivity (29). Moreover, after subcutaneous immunization of mice with influenza virus or VSV antigens, NK cells provide effective and specific protection against subsequent lethal infection (29). Similar to hapten-induced memory NK cells, influenza virus-induced memory NK cells also reside in the liver and are restricted to the CD49a^+^DX5^−^ NK cell population (44). In mouse models of vaccinia virus and herpes simplex virus 2 infection, NK cells exhibit an enhanced effector capacity and protection against secondary virus infection in a process specific to the priming virus (87, 88). More recently, antigen-specific memory NK cell responses have been observed in rhesus macaques after immunization with simian immunodeficiency virus (SIV) (45). In this study, NK cells from SIV-infected macaques showed increased cytotoxicity to Gag- and Env-pulsed DCs compared with that of NK cells from uninfected macaques, and this process was dependent on NKG2A and NKG2C (45). Furthermore, both splenic and hepatic NK cells from macaques vaccinated with adenovirus expressing SIV Gag or Env efficiently lysed antigen-matched, but not antigen-mismatched targets, and these antigen-specific NK cell responses were observed 5 years after vaccination (45), indicating the durability of these memory NK cell responses. These studies collectively provide compelling evidence that NK cells are endowed with immunological memory in different settings, but the specificities between NK cell receptors and cognate viral antigens remain unknown. Furthermore, it is unclear why memory NK cells induced by certain viruses are restricted to the liver, whereas there is no restriction on the tissue distribution of memory NK cells in other viral infections.

## Cytokine-Induced Memory-Like NK Cells

Several studies have indicated that NK cell activation by cytokines alone can induce the generation of NK cells with memory-like properties. In 2009, the Yokoyama group first reported that murine splenic NK cells with a history of combined cytokine activation display durable enhanced responses to restimulation (89). NK cells that were activated with IL-12, IL-15, and IL-18 *in vitro* and subsequently adoptively transferred to immunodeficient mice exhibited an enhanced capacity to produce IFN-γ upon restimulation with cytokines or *via* activating receptor engagement compared with pretreatment with IL-15 alone, and this enhanced response could be maintained for 12 weeks after adoptive transfer (89, 90). Notably, this memory-like property was shown to be intrinsic and was passed onto daughter generations that were not themselves exposed to cytokines *in vitro*, suggesting potential transcriptional or epigenetic changes in these memory-like NK cells (89, 90). Consistent with this hypothesis, a recent report showed that preactivation of mouse NK cells with combined cytokines led to demethylation of CNS1 at the *Ifng* locus (91), providing an explanation for the generation of these memory-like NK cells. Further studies are needed to investigate whether other epigenetic modifications are involved in this process.

In addition, memory-like NK cells induced by cytokine stimulation may exist *in vivo* during viral infections. A recent study showed that a portion of the NK cells from influenza-infected mice adoptively transferred into naïve recipients migrated to the bone marrow, where they exhibited significantly more division than those recovered from other tissues of the recipients and responded to subsequent influenza infection (92). Similar to cytokine-preactivated NK cells *in vitro*, these memory-like NK cells are not antigen specific, as these cells respond to the unrelated respiratory syncytial virus similar to influenza virus (92). Considering these findings together with the previous observation of elevated levels of the pro-inflammatory cytokines IL-12, IL-6, and IFN-γ in severe influenza patients (93), it is likely that cytokines drive these memory-like responses during influenza infections.

Follow-up studies from the Cerwenka group demonstrated that cytokine-preactivated NK cells maintained enhanced antitumor functions *in vivo*. The adoptive transfer of murine splenic NK cells pretreated with IL-12, IL-15, and IL-18, but not naïve or IL-15- or IL-2-pretreated NK cells, into tumor-bearing mice effectively reduced tumor growth, than that of when combined with radiation therapy (94). The memory-like NK cells in this model expressed high levels of CD25 (IL-2Rα), and their rapid proliferation *in vivo* was driven by IL-2 produced by CD4^+^ T cells. Moreover, the assistance provided by CD4^+^ T helper cells to memory-like NK cells is dependent on the presence of macrophages (91). However, the precise molecular mechanisms underlying the interactions between macrophages, T cells, and NK cells in this process remain uncharacterized.

The memory-like responses induced by previous cytokine stimulation have also been described in human NK cells. Similar to murine memory-like NK cells, human NK cells that are preactivated with IL-12, IL-15, and IL-18 and subsequently rest for several days display increased IFN-γ production upon restimulation with cytokines or target cells compared with control NK cells, and this enhanced activity is maintained following extensive cell division (94, 95). Both the CD56^bright^ and CD56^dim^ subsets of human NK cell exhibit memory-like properties, and in CD56^dim^ NK cells, enhanced IFN-γ production was also associated with a less mature phenotype, as evidenced by increased expression of CD94, NKG2A, NKG2C, and CD69 and a lack of CD57 and killer immunoglobulin-like receptor (KIR) expression among functional memory-like NK cells (94, 95). Moreover, the combination of CD16 cross-linking and cytokines resulted in robust enhancement of memory-like IFN-γ production, whereas NK cells preactivated through cross-linking CD16 alone did not elicit this effect (95), suggesting that activating receptor engagement could costimulate memory-like NK cell induction. Consistent with the results obtained from murine NK cells, human cytokine-preactivated NK cells were shown to display high CD25 expression, thus acquiring responsiveness to low doses of IL-2. Moreover, the functional competence of these cells was successfully supported by exogenous IL-2 after adoptive transfer into immunodeficient NOD-SCID-γ_c_^−/−^ (NSG) mice (96), suggesting that the administration of NK cells preactivated with combined cytokines *in vitro*, followed by IL-2 therapy *in vivo*, may be a useful therapeutic strategy for clinical trials.

## Therapeutic Potentials of NK Cell Memory

Considering the important role of NK cells in first-line defense against malignantly transformed cells, extensive efforts have been made to harness the antitumor effects of NK cells in the clinic for several decades (11). Despite significant progress in the field of NK cell-based immunotherapies, some factors limit the clinical efficacy of NK cells, such as their short lifespan and functional impairment (97). However, with recent advances in our understanding of NK cell memory, exploiting NK cells with memory-like properties might greatly increase the efficacy of these cells and pave the way for novel NK cell-based strategies for the clinical treatment of cancer (98).

The heightened antitumor responsiveness of cytokine-induced memory-like NK cells has been observed in both mice and humans. Consistent with the finding that murine cytokine-preactivated NK cells maintain enhanced antitumor activity *in vivo* after adoptive transfer (94), a preclinical study from the Fehniger group revealed that a single injection of human memory-like NK cells into NSG mice xenografted with K562 leukemia significantly reduced the leukemia burden and improved overall survival compared with control NK cells (99). Similarly, a separate study from the Cerwenka group also found effective control of solid tumor growth by cytokine-preactivated human NK cells in a melanoma xenograft model in NSG mice (91). The enhanced antitumor effects mediated by memory-like NK cells might result from their augmented cytotoxicity, high IFN-γ production capacity, and persistence in large numbers in the host (91).

The clinical application of allogeneic NK cells is promising for the treatment of leukemia and has attracted the interest of many investigators. KIR–ligand mismatch has a beneficial effect on donor NK cell alloreactivity against recipient leukemia (100–102). Moreover, many studies have shown that the adoptive transfer of alloreactive NK cells does not cause graft-versus-host disease (GVHD) (103–107) and instead suppresses GVHD (108). Although the development of GVHD after allogeneic NK cell adoptive transfer has been observed in several studies (109–111), an inadequate number of infused NK cells or other unknown factors during treatment might account for these consequences. Despite the advantages of NK cell-based immunotherapy, the inadequate persistence, expansion, and *in vivo* antitumor activity have limited the antileukemia effects of NK cells. To overcome these disadvantages, a Phase I clinical trial harnessing cytokine-induced memory-like NK cells has recently been initiated for patients with relapsed or refractory acute myeloid leukemia (AML) (99). In this clinical study, haploidentical donor-derived NK cells were preactivated with IL-12, IL-15, and IL-18 for 12–16 h, followed by washing and subsequent infusion into AML patients pretreated with the chemotherapy agents fludarabine/cyclophosphamide for lymphodepletion. After adoptive transfer, a low dose of IL-2 was administered for 2 weeks to support memory-like NK cell expansion and functionality. Tracking the donor memory-like NK cells in recipients revealed that the donor NK cells underwent expansion, with peak numbers in the blood and bone marrow being observed at 7–14 days after infusion, comprising >90% of blood NK cells (99). As expected, donor memory-like NK cells exhibit enhanced functionality after adoptive transfer, as evidenced by an increased frequency of IFN-γ-positive donor NK cells compared with that of recipient NK cells upon *ex vivo* restimulation with K562 leukemia cells (99). Notably, five of nine evaluable patients showed clinical responses, including four complete remissions, which compares favorably with previous studies utilizing purified NK cells without cytokine preactivation (112, 113). These results indicate the superiority of cytokine-induced memory-like NK cells in clinical applications for cancer therapy.

## Conclusion

Recent discoveries have shown that NK cells can remember prior exposure to haptens, viral antigens, or cytokine stimuli, leading to NK cell memory becoming a hot spot of current research. Moreover, emerging evidence has shown the clinical benefits of using NK cells with memory-like properties as a novel antitumor immunotherapy approach. Despite remarkable progress in this field, there are many questions that remain unanswered. For example, it is unclear whether NK cells recognize a variety of structurally distinct molecules with germ line-encoded receptors and why memory NK cells in certain models exhibit a restricted tissue distribution. Additionally, it is of interest to further investigate whether NK cell memory occurs in bacterial infections, autoimmune diseases, and cancer. Although other innate lymphoid cells (ILCs), such as ILC2s, also exhibit memory-like properties (114), it remains unknown whether these cells possess antigen-specific memory potential.

## Author Contributions

HP wrote the manuscript and ZT revised it.

## Conflict of Interest Statement

The authors declare that the research was conducted in the absence of any commercial or financial relationships that could be construed as a potential conflict of interest.

## References

[1] Plotkin SA. Correlates of protection induced by vaccination. Clin Vaccine Immunol (2010) 17(7):1055–65. 10.1128/CVI.00131-10 20463105 PMC2897268

[2] Sarkander J Hojyo S Tokoyoda K. Vaccination to gain humoral immune memory. Clin Transl Immunology (2016) 5(12):e120. 10.1038/cti.2016.81 28090322 PMC5192068

[3] Williams MA Bevan MJ. Effector and memory CTL differentiation. Annu Rev Immunol (2007) 25:171–92. 10.1146/annurev.immunol.25.022106.141548 17129182

[4] Chang JT Wherry EJ Goldrath AW. Molecular regulation of effector and memory T cell differentiation. Nat Immunol (2014) 15(12):1104–15. 10.1038/ni.3031 25396352 PMC4386685

[5] Liu J Cao X Cellular and molecular regulation of innate inflammatory responses. Cell Mol Immunol (2016) 13(6):711–21. 10.1038/cmi.2016.58 27818489 PMC5101451

[6] O’Sullivan TE Sun JC Lanier LL Natural killer cell memory. Immunity (2015) 43(4):634–45. 10.1016/j.immuni.2015.09.013 26488815 PMC4621966

[7] Kiessling R Klein E Wigzell H “Natural” killer cells in the mouse. I. Cytotoxic cells with specificity for mouse Moloney leukemia cells. Specificity and distribution according to genotype. Eur J Immunol (1975) 5(2):112–7. 10.1002/eji.1830050208 1234049

[8] Vivier E Raulet DH Moretta A Caligiuri MA Zitvogel L Lanier LL Innate or adaptive immunity? The example of natural killer cells. Science (2011) 331(6013):44–9. 10.1126/science.1198687 21212348 PMC3089969

[9] Martin-Fontecha A Thomsen LL Brett S Gerard C Lipp M Lanzavecchia A Induced recruitment of NK cells to lymph nodes provides IFN-gamma for T(H)1 priming. Nat Immunol (2004) 5(12):1260–5. 10.1038/ni1138 15531883

[10] Narni-Mancinelli E Ugolini S Vivier E. Tuning the threshold of natural killer cell responses. Curr Opin Immunol (2013) 25(1):53–8. 10.1016/j.coi.2012.11.005 23270590

[11] Guillerey C Huntington ND Smyth MJ. Targeting natural killer cells in cancer immunotherapy. Nat Immunol (2016) 17(9):1025–36. 10.1038/ni.3518 27540992

[12] Brandstadter JD Yang Y. Natural killer cell responses to viral infection. J Innate Immun (2011) 3(3):274–9. 10.1159/000324176 21411975 PMC3128146

[13] Liu S Chen L Zeng Y Si L Guo X Zhou J Suppressed expression of miR-378 targeting gzmb in NK cells is required to control dengue virus infection. Cell Mol Immunol (2016) 13(5):700–8. 10.1038/cmi.2015.52 26166761 PMC5037283

[14] Sun C Sun H Zhang C Tian Z. NK cell receptor imbalance and NK cell dysfunction in HBV infection and hepatocellular carcinoma. Cell Mol Immunol (2015) 12(3):292–302. 10.1038/cmi.2014.91 25308752 PMC4654321

[15] Zingoni A Sornasse T Cocks BG Tanaka Y Santoni A Lanier LL. Cross-talk between activated human NK cells and CD4+ T cells via OX40-OX40 ligand interactions. J Immunol (2004) 173(6):3716–24. 10.4049/jimmunol.173.6.3716 15356117

[16] Zingoni A Ardolino M Santoni A Cerboni C NKG2D and DNAM-1 activating receptors and their ligands in NK-T cell interactions: role in the NK cell-mediated negative regulation of T cell responses. Front Immunol (2012) 3:408 10.3389/fimmu.2012.00408 23316196 PMC3540764

[17] Moretta A. The dialogue between human natural killer cells and dendritic cells. Curr Opin Immunol (2005) 17(3):306–11. 10.1016/j.coi.2005.03.004 15886122

[18] Crouse J Xu HC Lang PA Oxenius A. NK cells regulating T cell responses: mechanisms and outcome. Trends Immunol (2015) 36(1):49–58. 10.1016/j.it.2014.11.001 25432489

[19] Cerwenka A Lanier LL. Natural killer cell memory in infection, inflammation and cancer. Nat Rev Immunol (2016) 16(2):112–23. 10.1038/nri.2015.9 26806484

[20] O’Leary JG Goodarzi M Drayton DL von Andrian UH. T cell- and B cell-independent adaptive immunity mediated by natural killer cells. Nat Immunol (2006) 7(5):507–16. 10.1038/ni1332 16617337

[21] Marchal G Seman M Milon G Truffa-Bachi P Zilberfarb V. Local adoptive transfer of skin delayed-type hypersensitivity initiated by a single T lymphocyte. J Immunol (1982) 129(3):954–8. 6980927

[22] Hochgeschwender U Simon HG Weltzien HU Bartels F Becker A Epplen JT. Dominance of one T-cell receptor in the H-2Kb/TNP response. Nature (1987) 326(6110):307–9. 10.1038/326307a0 3493439

[23] Askenase PW. Yes T cells, but three different T cells (alphabeta, gammadelta and NK T cells), and also B-1 cells mediate contact sensitivity. Clin Exp Immunol (2001) 125(3):345–50. 10.1046/j.1365-2249.2001.01619.x 11531940 PMC1906150

[24] Yokozeki H Watanabe K Igawa K Miyazaki Y Katayama I Nishioka K. Gammadelta T cells assist alphabeta T cells in the adoptive transfer of contact hypersensitivity to para-phenylenediamine. Clin Exp Immunol (2001) 125(3):351–9. 10.1046/j.1365-2249.2001.01570.x 11531941 PMC1906141

[25] Tsuji RF Szczepanik M Kawikova I Paliwal V Campos RA Itakura A B cell-dependent T cell responses: IgM antibodies are required to elicit contact sensitivity. J Exp Med (2002) 196(10):1277–90. 10.1084/jem.20020649 12438420 PMC2193992

[26] Askenase PW Itakura A Leite-de-Moraes MC Lisbonne M Roongapinun S Goldstein DR TLR-dependent IL-4 production by invariant Valpha14+Jalpha18+ NKT cells to initiate contact sensitivity in vivo. J Immunol (2005) 175(10):6390–401. 10.4049/jimmunol.175.10.6390 16272291

[27] Yokoyama WM Contact hypersensitivity: not just T cells! Nat Immunol (2006) 7(5):437–9. 10.1038/ni0506-437 16622426

[28] Majewska-Szczepanik M Paust S von Andrian UH Askenase PW Szczepanik M Natural killer cell-mediated contact sensitivity develops rapidly and depends on interferon-alpha, interferon-gamma and interleukin-12. Immunology (2013) 140(1):98–110. 10.1111/imm.12120 23659714 PMC3809710

[29] Paust S Gill HS Wang BZ Flynn MP Moseman EA Senman B Critical role for the chemokine receptor CXCR6 in NK cell-mediated antigen-specific memory of haptens and viruses. Nat Immunol (2010) 11(12):1127–35. 10.1038/ni.1953 20972432 PMC2982944

[30] Knolle PA Wohlleber D. Immunological functions of liver sinusoidal endothelial cells. Cell Mol Immunol (2016) 13(3):347–53. 10.1038/cmi.2016.5 27041636 PMC4856811

[31] Peng H Jiang X Chen Y Sojka DK Wei H Gao X Liver-resident NK cells confer adaptive immunity in skin-contact inflammation. J Clin Invest (2013) 123(4):1444–56. 10.1172/JCI66381 23524967 PMC3613925

[32] van den Boorn JG Picavet DI van Swieten PF van Veen HA Konijnenberg D van Veelen PA Skin-depigmenting agent monobenzone induces potent T-cell autoimmunity toward pigmented cells by tyrosinase haptenation and melanosome autophagy. J Invest Dermatol (2011) 131(6):1240–51. 10.1038/jid.2011.16 21326294

[33] van den Boorn JG Jakobs C Hagen C Renn M Luiten RM Melief CJ Inflammasome-dependent induction of adaptive NK cell memory. Immunity (2016) 44(6):1406–21. 10.1016/j.immuni.2016.05.008 27287410

[34] Peng H Wisse E Tian Z Liver natural killer cells: subsets and roles in liver immunity. Cell Mol Immunol (2016) 13(3):328–36. 10.1038/cmi.2015.96 26639736 PMC4856807

[35] Sojka DK Plougastel-Douglas B Yang L Pak-Wittel MA Artyomov MN Ivanova Y Tissue-resident natural killer (NK) cells are cell lineages distinct from thymic and conventional splenic NK cells. Elife (2014) 3:e01659. 10.7554/eLife.01659 24714492 PMC3975579

[36] Daussy C Faure F Mayol K Viel S Gasteiger G Charrier E T-bet and Eomes instruct the development of two distinct natural killer cell lineages in the liver and in the bone marrow. J Exp Med (2014) 211(3):563–77. 10.1084/jem.20131560 24516120 PMC3949572

[37] Mackay LK Minnich M Kragten NA Liao Y Nota B Seillet C Hobit and Blimp1 instruct a universal transcriptional program of tissue residency in lymphocytes. Science (2016) 352(6284):459–63. 10.1126/science.aad2035 27102484

[38] Scholz F Naik S Sutterwala FS Kaplan DH. Langerhans cells suppress CD49a+ NK cell-mediated skin inflammation. J Immunol (2015) 195(5):2335–42. 10.4049/jimmunol.1500935 26209621 PMC4546924

[39] Zhang LH Shin JH Haggadone MD Sunwoo JB. The aryl hydrocarbon receptor is required for the maintenance of liver-resident natural killer cells. J Exp Med (2016) 213(11):2249–57. 10.1084/jem.20151998 27670593 PMC5068230

[40] Stegmann KA Robertson F Hansi N Gill U Pallant C Christophides T CXCR6 marks a novel subset of T-bet(lo)Eomes(hi) natural killer cells residing in human liver. Sci Rep (2016) 6:26157. 10.1038/srep26157 27210614 PMC4876507

[41] Hudspeth K Donadon M Cimino M Pontarini E Tentorio P Preti M Human liver-resident CD56(bright)/CD16(neg) NK cells are retained within hepatic sinusoids via the engagement of CCR5 and CXCR6 pathways. J Autoimmun (2016) 66:40–50. 10.1016/j.jaut.2015.08.011 26330348 PMC4718768

[42] Aw Yeang HX Piersma SJ Lin Y Yang L Malkova ON Miner C Cutting edge: human CD49e- NK cells are tissue resident in the liver. J Immunol (2017) 198(4):1417–22. 10.4049/jimmunol.1601818 28093522 PMC5296254

[43] Sun JC Beilke JN Lanier LL. Adaptive immune features of natural killer cells. Nature (2009) 457(7229):557–61. 10.1038/nature07665 19136945 PMC2674434

[44] Li T Wang J Wang Y Chen Y Wei H Sun R Respiratory influenza virus infection induces memory-like liver NK cells in mice. J Immunol (2017) 198(3):1242–52. 10.4049/jimmunol.1502186 28031334

[45] Reeves RK Li H Jost S Blass E Schafer JL Varner V Antigen-specific NK cell memory in rhesus macaques. Nat Immunol (2015) 16(9):927–32. 10.1038/ni.3227 26193080 PMC4545390

[46] Lopez-Verges S Milush JM Schwartz BS Pando MJ Jarjoura J York VA Expansion of a unique CD57(+)NKG2Chi natural killer cell subset during acute human cytomegalovirus infection. Proc Natl Acad Sci U S A (2011) 108(36):14725–32. 10.1073/pnas.1110900108 21825173 PMC3169160

[47] Beziat V Dalgard O Asselah T Halfon P Bedossa P Boudifa A CMV drives clonal expansion of NKG2C+ NK cells expressing self-specific KIRs in chronic hepatitis patients. Eur J Immunol (2012) 42(2):447–57. 10.1002/eji.201141826 22105371

[48] Slavuljica I Kvestak D Huszthy PC Kosmac K Britt WJ Jonjic S Immunobiology of congenital cytomegalovirus infection of the central nervous system-the murine cytomegalovirus model. Cell Mol Immunol (2015) 12(2):180–91. 10.1038/cmi.2014.51 25042632 PMC4654296

[49] Brown MG Dokun AO Heusel JW Smith HR Beckman DL Blattenberger EA Vital involvement of a natural killer cell activation receptor in resistance to viral infection. Science (2001) 292(5518):934–7. 10.1126/science.1060042 11340207

[50] Arase H Mocarski ES Campbell AE Hill AB Lanier LL. Direct recognition of cytomegalovirus by activating and inhibitory NK cell receptors. Science (2002) 296(5571):1323–6. 10.1126/science.1070884 11950999

[51] Dokun AO Kim S Smith HR Kang HS Chu DT Yokoyama WM. Specific and nonspecific NK cell activation during virus infection. Nat Immunol (2001) 2(10):951–6. 10.1038/ni714 11550009

[52] Bubic I Wagner M Krmpotic A Saulig T Kim S Yokoyama WM Gain of virulence caused by loss of a gene in murine cytomegalovirus. J Virol (2004) 78(14):7536–44. 10.1128/JVI.78.14.7536-7544.2004 15220428 PMC434107

[53] Sun JC Beilke JN Lanier LL. Immune memory redefined: characterizing the longevity of natural killer cells. Immunol Rev (2010) 236:83–94. 10.1111/j.1600-065X.2010.00900.x 20636810 PMC2907527

[54] Nabekura T Kanaya M Shibuya A Fu G Gascoigne NR Lanier LL. Costimulatory molecule DNAM-1 is essential for optimal differentiation of memory natural killer cells during mouse cytomegalovirus infection. Immunity (2014) 40(2):225–34. 10.1016/j.immuni.2013.12.011 24440149 PMC3943894

[55] Karo JM Schatz DG Sun JC. The RAG recombinase dictates functional heterogeneity and cellular fitness in natural killer cells. Cell (2014) 159(1):94–107. 10.1016/j.cell.2014.08.026 25259923 PMC4371485

[56] Hendricks DW Min-Oo G Lanier LL. Sweet is the memory of past troubles: NK cells remember. Curr Top Microbiol Immunol (2016) 395:147–71. 10.1007/82_2015_447 26099194

[57] Sun JC Madera S Bezman NA Beilke JN Kaplan MH Lanier LL. Proinflammatory cytokine signaling required for the generation of natural killer cell memory. J Exp Med (2012) 209(5):947–54. 10.1084/jem.20111760 22493516 PMC3348098

[58] Madera S Sun JC. Cutting edge: stage-specific requirement of IL-18 for antiviral NK cell expansion. J Immunol (2015) 194(4):1408–12. 10.4049/jimmunol.1402001 25589075 PMC4323636

[59] Nabekura T Girard JP Lanier LL. IL-33 receptor ST2 amplifies the expansion of NK cells and enhances host defense during mouse cytomegalovirus infection. J Immunol (2015) 194(12):5948–52. 10.4049/jimmunol.1500424 25926677 PMC4458425

[60] Firth MA Madera S Beaulieu AM Gasteiger G Castillo EF Schluns KS Nfil3-independent lineage maintenance and antiviral response of natural killer cells. J Exp Med (2013) 210(13):2981–90. 10.1084/jem.20130417 24277151 PMC3865482

[61] Zawislak CL Beaulieu AM Loeb GB Karo J Canner D Bezman NA Stage-specific regulation of natural killer cell homeostasis and response against viral infection by microRNA-155. Proc Natl Acad Sci U S A (2013) 110(17):6967–72. 10.1073/pnas.1304410110 23572582 PMC3637707

[62] Beaulieu AM Zawislak CL Nakayama T Sun JC. The transcription factor Zbtb32 controls the proliferative burst of virus-specific natural killer cells responding to infection. Nat Immunol (2014) 15(6):546–53. 10.1038/ni.2876 24747678 PMC4404304

[63] Min-Oo G Bezman NA Madera S Sun JC Lanier LL. Proapoptotic Bim regulates antigen-specific NK cell contraction and the generation of the memory NK cell pool after cytomegalovirus infection. J Exp Med (2014) 211(7):1289–96. 10.1084/jem.20132459 24958849 PMC4076589

[64] O’Sullivan TE Johnson LR Kang HH Sun JC. BNIP3- and BNIP3L-mediated mitophagy promotes the generation of natural killer cell memory. Immunity (2015) 43(2):331–42. 10.1016/j.immuni.2015.07.012 26253785 PMC5737626

[65] Puleston DJ Zhang H Powell TJ Lipina E Sims S Panse I Autophagy is a critical regulator of memory CD8(+) T cell formation. Elife (2014) 3:e03706. 10.7554/eLife.03706 25385531 PMC4225493

[66] Wills MR Poole E Lau B Krishna B Sinclair JH. The immunology of human cytomegalovirus latency: could latent infection be cleared by novel immunotherapeutic strategies? Cell Mol Immunol (2015) 12(2):128–38. 10.1038/cmi.2014.75 25132454 PMC4654298

[67] McCormick AL Mocarski ES. The immunological underpinnings of vaccinations to prevent cytomegalovirus disease. Cell Mol Immunol (2015) 12(2):170–9. 10.1038/cmi.2014.120 25544503 PMC4654290

[68] Guma M Angulo A Vilches C Gomez-Lozano N Malats N Lopez-Botet M. Imprint of human cytomegalovirus infection on the NK cell receptor repertoire. Blood (2004) 104(12):3664–71. 10.1182/blood-2004-05-2058 15304389

[69] Monsivais-Urenda A Noyola-Cherpitel D Hernandez-Salinas A Garcia-Sepulveda C Romo N Baranda L Influence of human cytomegalovirus infection on the NK cell receptor repertoire in children. Eur J Immunol (2010) 40(5):1418–27. 10.1002/eji.200939898 20201038

[70] Noyola DE Alarcon A Noguera-Julian A Muntasell A Munoz-Almagro C Garcia J Dynamics of the NK-cell subset redistribution induced by cytomegalovirus infection in preterm infants. Hum Immunol (2015) 76(2–3):118–23. 10.1016/j.humimm.2015.01.017 25636568

[71] Guma M Cabrera C Erkizia I Bofill M Clotet B Ruiz L Human cytomegalovirus infection is associated with increased proportions of NK cells that express the CD94/NKG2C receptor in aviremic HIV-1-positive patients. J Infect Dis (2006) 194(1):38–41. 10.1086/504719 16741880

[72] Bjorkstrom NK Lindgren T Stoltz M Fauriat C Braun M Evander M Rapid expansion and long-term persistence of elevated NK cell numbers in humans infected with hantavirus. J Exp Med (2011) 208(1):13–21. 10.1084/jem.20100762 21173105 PMC3023129

[73] Guma M Budt M Saez A Brckalo T Hengel H Angulo A Expansion of CD94/NKG2C+ NK cells in response to human cytomegalovirus-infected fibroblasts. Blood (2006) 107(9):3624–31. 10.1182/blood-2005-09-3682 16384928

[74] Kuijpers TW Baars PA Dantin C van den Burg M van Lier RA Roosnek E Human NK cells can control CMV infection in the absence of T cells. Blood (2008) 112(3):914–5. 10.1182/blood-2008-05-157354 18650467

[75] Muccio L Bertaina A Falco M Pende D Meazza R Lopez-Botet M Analysis of memory-like natural killer cells in human cytomegalovirus-infected children undergoing alphabeta+T and B cell-depleted hematopoietic stem cell transplantation for hematological malignancies. Haematologica (2016) 101(3):371–81. 10.3324/haematol.2015.134155 26659918 PMC4815729

[76] Foley B Cooley S Verneris MR Pitt M Curtsinger J Luo X Cytomegalovirus reactivation after allogeneic transplantation promotes a lasting increase in educated NKG2C+ natural killer cells with potent function. Blood (2012) 119(11):2665–74. 10.1182/blood-2011-10-386995 22180440 PMC3311280

[77] Foley B Cooley S Verneris MR Curtsinger J Luo X Waller EK Human cytomegalovirus (CMV)-induced memory-like NKG2C(+) NK cells are transplantable and expand in vivo in response to recipient CMV antigen. J Immunol (2012) 189(10):5082–8. 10.4049/jimmunol.1201964 23077239 PMC3490031

[78] Rolle A Pollmann J Ewen EM Le VT Halenius A Hengel H IL-12-producing monocytes and HLA-E control HCMV-driven NKG2C+ NK cell expansion. J Clin Invest (2014) 124(12):5305–16. 10.1172/JCI77440 25384219 PMC4348979

[79] Luetke-Eversloh M Hammer Q Durek P Nordstrom K Gasparoni G Pink M Human cytomegalovirus drives epigenetic imprinting of the IFNG locus in NKG2Chi natural killer cells. PLoS Pathog (2014) 10(10):e1004441. 10.1371/journal.ppat.1004441 25329659 PMC4199780

[80] Hwang I Zhang T Scott JM Kim AR Lee T Kakarla T Identification of human NK cells that are deficient for signaling adaptor FcRgamma and specialized for antibody-dependent immune functions. Int Immunol (2012) 24(12):793–802. 10.1093/intimm/dxs080 22962434 PMC3621379

[81] Zhang T Scott JM Hwang I Kim S Cutting edge: antibody-dependent memory-like NK cells distinguished by FcRgamma deficiency. J Immunol (2013) 190(4):1402–6. 10.4049/jimmunol.1203034 23345329 PMC3623944

[82] Schlums H Cichocki F Tesi B Theorell J Beziat V Holmes TD Cytomegalovirus infection drives adaptive epigenetic diversification of NK cells with altered signaling and effector function. Immunity (2015) 42(3):443–56. 10.1016/j.immuni.2015.02.008 25786176 PMC4612277

[83] Lee J Zhang T Hwang I Kim A Nitschke L Kim M Epigenetic modification and antibody-dependent expansion of memory-like NK cells in human cytomegalovirus-infected individuals. Immunity (2015) 42(3):431–42. 10.1016/j.immuni.2015.02.013 25786175 PMC4537797

[84] Noyola DE Fortuny C Muntasell A Noguera-Julian A Munoz-Almagro C Alarcon A Influence of congenital human cytomegalovirus infection and the NKG2C genotype on NK-cell subset distribution in children. Eur J Immunol (2012) 42(12):3256–66. 10.1002/eji.201242752 22965785

[85] Liu LL Landskron J Ask EH Enqvist M Sohlberg E Traherne JA Critical role of CD2 Co-stimulation in adaptive natural killer cell responses revealed in NKG2C-deficient humans. Cell Rep (2016) 15(5):1088–99. 10.1016/j.celrep.2016.04.005 27117418 PMC4858565

[86] Della Chiesa M Falco M Bertaina A Muccio L Alicata C Frassoni F Human cytomegalovirus infection promotes rapid maturation of NK cells expressing activating killer Ig-like receptor in patients transplanted with NKG2C−/− umbilical cord blood. J Immunol (2014) 192(4):1471–9. 10.4049/jimmunol.1302053 24442432

[87] Gillard GO Bivas-Benita M Hovav AH Grandpre LE Panas MW Seaman MS Thy1+ NK [corrected] cells from vaccinia virus-primed mice confer protection against vaccinia virus challenge in the absence of adaptive lymphocytes. PLoS Pathog (2011) 7(8):e1002141. 10.1371/journal.ppat.1002141 21829360 PMC3150274

[88] Abdul-Careem MF Lee AJ Pek EA Gill N Gillgrass AE Chew MV Genital HSV-2 infection induces short-term NK cell memory. PLoS One (2012) 7(3):e32821. 10.1371/journal.pone.0032821 22457721 PMC3310819

[89] Cooper MA Elliott JM Keyel PA Yang L Carrero JA Yokoyama WM. Cytokine-induced memory-like natural killer cells. Proc Natl Acad Sci U S A (2009) 106(6):1915–9. 10.1073/pnas.0813192106 19181844 PMC2644138

[90] Keppel MP Yang L Cooper MA. Murine NK cell intrinsic cytokine-induced memory-like responses are maintained following homeostatic proliferation. J Immunol (2013) 190(9):4754–62. 10.4049/jimmunol.1201742 23530145 PMC3633638

[91] Ni J Holsken O Miller M Hammer Q Luetke-Eversloh M Romagnani C Adoptively transferred natural killer cells maintain long-term antitumor activity by epigenetic imprinting and CD4+ T cell help. Oncoimmunology (2016) 5(9):e1219009. 10.1080/2162402X.2016.1219009 27757318 PMC5048776

[92] van Helden MJ de Graaf N Boog CJ Topham DJ Zaiss DM Sijts AJ. The bone marrow functions as the central site of proliferation for long-lived NK cells. J Immunol (2012) 189(5):2333–7. 10.4049/jimmunol.1200008 22821961 PMC3427014

[93] Heltzer ML Coffin SE Maurer K Bagashev A Zhang Z Orange JS Immune dysregulation in severe influenza. J Leukoc Biol (2009) 85(6):1036–43. 10.1189/jlb.1108710 19276177 PMC2698588

[94] Ni J Miller M Stojanovic A Garbi N Cerwenka A. Sustained effector function of IL-12/15/18-preactivated NK cells against established tumors. J Exp Med (2012) 209(13):2351–65. 10.1084/jem.20120944 23209317 PMC3526364

[95] Romee R Schneider SE Leong JW Chase JM Keppel CR Sullivan RP Cytokine activation induces human memory-like NK cells. Blood (2012) 120(24):4751–60. 10.1182/blood-2012-04-419283 22983442 PMC3520618

[96] Leong JW Chase JM Romee R Schneider SE Sullivan RP Cooper MA Preactivation with IL-12, IL-15, and IL-18 induces CD25 and a functional high-affinity IL-2 receptor on human cytokine-induced memory-like natural killer cells. Biol Blood Marrow Transplant (2014) 20(4):463–73. 10.1016/j.bbmt.2014.01.006 24434782 PMC3959288

[97] Rezvani K Rouce RH. The application of natural killer cell immunotherapy for the treatment of cancer. Front Immunol (2015) 6:578. 10.3389/fimmu.2015.00578 26635792 PMC4648067

[98] Fehniger TA Cooper MA. Harnessing NK cell memory for cancer immunotherapy. Trends Immunol (2016) 37(12):877–88. 10.1016/j.it.2016.09.005 27773685 PMC5135622

[99] Romee R Rosario M Berrien-Elliott MM Wagner JA Jewell BA Schappe T Cytokine-induced memory-like natural killer cells exhibit enhanced responses against myeloid leukemia. Sci Transl Med (2016) 8(357):357ra123. 10.1126/scitranslmed.aaf2341 27655849 PMC5436500

[100] Cooley S Weisdorf DJ Guethlein LA Klein JP Wang T Le CT Donor selection for natural killer cell receptor genes leads to superior survival after unrelated transplantation for acute myelogenous leukemia. Blood (2010) 116(14):2411–9. 10.1182/blood-2010-05-283051 20581313 PMC2953880

[101] Cooley S Trachtenberg E Bergemann TL Saeteurn K Klein J Le CT Donors with group B KIR haplotypes improve relapse-free survival after unrelated hematopoietic cell transplantation for acute myelogenous leukemia. Blood (2009) 113(3):726–32. 10.1182/blood-2008-07-171926 18945962 PMC2628378

[102] Venstrom JM Pittari G Gooley TA Chewning JH Spellman S Haagenson M HLA-C-dependent prevention of leukemia relapse by donor activating KIR2DS1. N Engl J Med (2012) 367(9):805–16. 10.1056/NEJMoa1200503 22931314 PMC3767478

[103] Ruggeri L Capanni M Urbani E Perruccio K Shlomchik WD Tosti A Effectiveness of donor natural killer cell alloreactivity in mismatched hematopoietic transplants. Science (2002) 295(5562):2097–100. 10.1126/science.1068440 11896281

[104] Passweg JR Tichelli A Meyer-Monard S Heim D Stern M Kuhne T Purified donor NK-lymphocyte infusion to consolidate engraftment after haploidentical stem cell transplantation. Leukemia (2004) 18(11):1835–8. 10.1038/sj.leu.2403524 15457184

[105] Choi I Yoon SR Park SY Kim H Jung SJ Jang YJ Donor-derived natural killer cells infused after human leukocyte antigen-haploidentical hematopoietic cell transplantation: a dose-escalation study. Biol Blood Marrow Transplant (2014) 20(5):696–704. 10.1016/j.bbmt.2014.01.031 24525278

[106] Jaiswal SR Zaman S Nedunchezhian M Chakrabarti A Bhakuni P Ahmed M CD56-enriched donor cell infusion after post-transplantation cyclophosphamide for haploidentical transplantation of advanced myeloid malignancies is associated with prompt reconstitution of mature natural killer cells and regulatory T cells with reduced incidence of acute graft versus host disease: a pilot study. Cytotherapy (2017) 19(4):531–42. 10.1016/j.jcyt.2016.12.006 28131632

[107] Simonetta F Alvarez M Negrin RS. Natural killer cells in graft-versus-host-disease after allogeneic hematopoietic cell transplantation. Front Immunol (2017) 8:465. 10.3389/fimmu.2017.00465 28487696 PMC5403889

[108] Olson JA Leveson-Gower DB Gill S Baker J Beilhack A Negrin RS. NK cells mediate reduction of GVHD by inhibiting activated, alloreactive T cells while retaining GVT effects. Blood (2010) 115(21):4293–301. 10.1182/blood-2009-05-222190 20233969 PMC2879101

[109] Yoon SR Lee YS Yang SH Ahn KH Lee JH Kim DY Generation of donor natural killer cells from CD34(+) progenitor cells and subsequent infusion after HLA-mismatched allogeneic hematopoietic cell transplantation: a feasibility study. Bone Marrow Transplant (2010) 45(6):1038–46. 10.1038/bmt.2009.304 19881555

[110] Stern M Passweg JR Meyer-Monard S Esser R Tonn T Soerensen J Pre-emptive immunotherapy with purified natural killer cells after haploidentical SCT: a prospective phase II study in two centers. Bone Marrow Transplant (2013) 48(3):433–8. 10.1038/bmt.2012.162 22941380

[111] Lee DA Denman CJ Rondon G Woodworth G Chen J Fisher T Haploidentical natural killer cells infused before allogeneic stem cell transplantation for myeloid malignancies: a phase I trial. Biol Blood Marrow Transplant (2016) 22(7):1290–8. 10.1016/j.bbmt.2016.04.009 27090958 PMC4905771

[112] Bachanova V Cooley S Defor TE Verneris MR Zhang B McKenna DH Clearance of acute myeloid leukemia by haploidentical natural killer cells is improved using IL-2 diphtheria toxin fusion protein. Blood (2014) 123(25):3855–63. 10.1182/blood-2013-10-532531 24719405 PMC4064329

[113] Curti A Ruggeri L D’Addio A Bontadini A Dan E Motta MR Successful transfer of alloreactive haploidentical KIR ligand-mismatched natural killer cells after infusion in elderly high risk acute myeloid leukemia patients. Blood (2011) 118(12):3273–9. 10.1182/blood-2011-01-329508 21791425

[114] Martinez-Gonzalez I Matha L Steer CA Ghaedi M Poon GF Takei F. Allergen-experienced group 2 innate lymphoid cells acquire memory-like properties and enhance allergic lung inflammation. Immunity (2016) 45(1):198–208. 10.1016/j.immuni.2016.06.017 27421705

